# Chemico-pharmacological evaluation of the methanolic leaf extract of *Catharanthus ovalis*: GC–MS/MS, *in vivo*, *in vitro*, and *in silico* approaches

**DOI:** 10.3389/fphar.2024.1347069

**Published:** 2024-03-01

**Authors:** Saimon Shahriar, Samia Akter Shermin, Hasin Hasnat, Faisal Hossain, Aixia Han, Peiwu Geng, Safaet Alam, Abdullah Al Mamun

**Affiliations:** ^1^ Department of Pharmacy, School of Pharmaceutical Sciences, State University of Bangladesh, Dhaka, Bangladesh; ^2^ Central Laboratory of The Sixth Affiliated Hospital of Wenzhou Medical University, Lishui People’s Hospital, Lishui, Zhejiang, China; ^3^ Drugs and Toxins Research Division, BCSIR Laboratories Rajshahi, Bangladesh Council of Scientific and Industrial Research, Rajshahi, Bangladesh

**Keywords:** phytochemical, GC–MS/MS, *Catharanthus ovalis*, membrane stabilizing, analgesic, thrombolytic, *in silico*, *in vitro*

## Abstract

**Introduction:** Natural plant-based medicines have gained popularity recently as a major source of inventive, risk-free, and more potent secondary bioactive compounds with medicinal potential. *Catharanthus ovalis* is a perennial shrub containing various indole alkaloids cultivated extensively for local medical purposes.

**Methods:** This research is conducted to identify the phytocompounds present in the leaves of *C. ovalis* and its central and peripheral analgesic, thrombolytic, and membrane-stabilizing activities through tail immersion, acetic acid-induced writhing, human blood clot lysis, and erythrocyte lysis by heat and hypotonic solution methods, respectively.

**Results and discussion:** A total of 39 compounds were identified using GC–MS/MS techniques, including hexadecanoic acid, methyl ester (56.749%), methyl stearate (29.782%), carvacrol and its TBDMS derivative (12.586%), and 9-octadecenoic acid, methyl ester, (E)-] (9.297%) presented in high quantity. The highest tail immersion latency was observed for the 600 mg/kg extract of *C. ovalis* crude extract. Both 400 and 600 mg/kg doses of *C. ovalis* crude extract exhibited prominent peripheral analgesic activity. The maximum thrombolytic effect was observed by DCM soluble fraction extract by inhibiting 54.87% of the clot. However, the aqueous-soluble fraction of this extract manifested an excellent membrane-stabilizing effect by showing 73.98% and 87.51% hemolysis against heat- and hypotonic-induced hemolysis, respectively. Some of the compounds were identified as active agents against different receptors related to these diseases, which supported the findings of *in vitro* and *in vivo* tests.

**Conclusion:** Further investigation needs to be conducted to specify and identify the exact mechanism of action of these compounds.

## Graphical Abstract

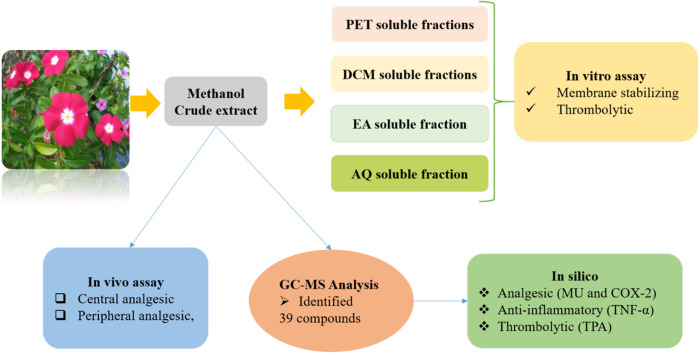

## 1 Introduction

Since ancient times, plants have been used as excellent sources of medicines to treat many illnesses. Recently, natural plant-based products have drawn attention as a key source of creative, safer, and more potent secondary bioactive metabolites with therapeutic potential. Nearly 80% of all pharmaceuticals are either wholly or partially derived from plants (Obonti et al., 2021). According to the World Health Organization (WHO), 80% of people utilize herbal medicines as part of their primary healthcare. Plant-based treatments are anticipated to make up to 25% of all drugs in developed nations such as the United States while making up to 80% of all drugs in rapidly growing nations such as India and China. Even in the modern period, plants are still a potential source of medication for serious illnesses such as cancer, oxidative stress, cancer, diarrhea, depression, fever, and thrombosis (Islam et al., 2022). It has been demonstrated that *in vitro* screening techniques can offer essential initial observations required to choose crude plant extracts with potentially beneficial features for additional chemical and pharmacological research (Mathekga and Meyer, 1998).

*Catharanthus* is a genus of flowering plants of the Apocynaceae (dogbane) family (Tolambiya and Mathur, 2016). It has glossy opposing leaves, and its stunning five-petaled flowers come in a range of pink, white, and purple hues and can be distinguished by its evergreen, herbaceous, or subshrubby habit (Mishra and Verma et al., 2017). Typically, there are eight species in this genus, seven of which are native to Madagascar, namely, *Catharanthus coriaceus, Catharanthus lanceus, Catharanthus longifolius, C. ovalis, Catharanthus roseus, Catharanthus scitulus,* and *Catharanthus trichophyllus*, and one of which, namely, *Catharanthus pusillus*, has been reported from India (Alam et al., 2017). Historically, the Caribbean native plant family Apocynaceae has been utilized for a variety of medical conditions; especially, European herbalists used plants of these families for diabetes and illnesses as diverse as headaches, as well as for wound healing (Kabesh et al., 2015).

However, the *Catharanthus* genus is well known for its diverse phytochemical composition, especially for the high concentrations of alkaloids, terpenoids, and flavonoids. Vinblastine and vincristine are two of the most noteworthy alkaloids and have received much attention for their powerful anticancer properties. In addition, several other bioactive substances have been discovered, contributing to a variety of pharmacological actions (Pham et al., 2020). *Catharanthus roseus*, the most common species of the *Catharanthus* genus rooted in traditional folklore, is revered for its diverse applications in treating burns, rheumatism, and menstrual disorders and its potential in managing hypertension, diabetes, and cancer and supporting uterine contractions (Chaturvedi et al., 2022; De et al., 2022).

*Catharanthus ovalis* Markgr., a perennial plant, typically grows in dry shrubland or desert biomes and can grow up to 40 cm in height (Tropical Plants Database, 2023). Although indigenous to southern Madagascar and Africa, it is cultivated extensively for local medical purposes. Historically, the plant’s pure extract was consumed as a trial poison. Additionally, worms are expelled using an extract of the aerial sections. Indole alkaloids, including many bisindole alkaloids, are abundant in this species. On the other hand, it comprises several pharmacologically effective chemicals such as leurosine, vindoline, vindolinine, coronaridine, catharanthine, and vinblastine (Tropical Plants Database, 2023). Despite its recognized medicinal potency, there is a notable absence of comprehensive pharmacological research on this species, limiting our understanding of its full therapeutic potential and applications. The purpose of this investigation was to identify the phytochemicals present in the *C. ovalis* leaf tissue through GC–MS/MS techniques. Additionally, this study used *in vivo* and *in vitro* methodologies to examine several biological functions of the leaf extract. However, the *in silico* approach was adopted for the preliminary screening of the identified phytochemicals responsible for those biological activities.

## 2 Materials and methods

### 2.1 Plant material

*Catharanthus ovalis* Markgr. was collected from Sylhet, Bangladesh, and was identified by the Bangladesh National Herbarium with an accession number of DACB 88049. The plant is prepared and preserved in the phytochemical research laboratory of the Department of Pharmacy at the State University of Bangladesh.

### 2.2 Preparation of extract and subsequent fractions

The *C. ovalis* leaf material was taken from the wild, shade-dried, and turned into a powder using a mechanical grinder. The needed amount of leaf powder was weighed, transferred to a flask, totally submerged in methanol, incubated for 15 days, and then, filtered. Following that, a rotary evaporator was used to concentrate the filtered extract (Islam et al., 2020). The concentrated extract contains both polar and nonpolar plant material components, and 2 µL of the sample solution was used in GC–MS/MS for chemical analysis.

Solvent–solvent partitioning was conducted using the process described by VanWagenen et al. (1993). A measure of 5 g of the crude methanolic extract of *C. ovalis* (COCME) was dissolved in 10% aqueous methanol and then extracted sequentially with petroleum ether, dichloromethane, ethyl acetate, and finally, water to obtain four respective fractions: petroleum ether-soluble fraction (COPESF), dichloromethane-soluble fraction (CODMSF), ethyl acetate-soluble fraction (COEASF), and aqueous-soluble fraction (COASF). All the fractions were evaporated to dryness and were used for further analysis.

### 2.3 Phytochemical analysis

#### 2.3.1 GC–MS/MS analysis and the identification of phytocompounds

A GCMS-TQ8040 GC–MS/MS (Shimadzu, Japan) system with an Elite-5MS (5% diphenyl/95% dimethyl polysiloxane) and a fused capillary column (30 0.25 m ID 0.25 m df) was used to conduct the GC–MS/MS analysis of the methanolic extract of *C. ovalis*. The electron ionization system employed for GC–MS/MS detection operated in an electron impact mode with an ionization energy of 70 eV. An electron ionization system with an ionization energy of 70 eV was run in an electron impact mode for GC–MS/MS detection. As a carrier gas, helium gas (99.999%) was used with an injection volume of 2 µL and a constant flow rate of 1 mL/min (a split ratio of 10:1). The oven temperature was programmed to start at 50°C (isothermal for 1 min), increase by 15°C/min to 200°C, then 5°C/min to 300°C, and end with a 7 min isothermal at 300°C. The injector temperature was kept at 250°C. At a scan speed of 2000, mass spectra were collected at 70ev. The entire running time for the GC/MS was 36 min, and the solvent delay was 3.50 min. The spectrum was measured between 50 and 600 m/z. Comparing each component’s average peak area to the total areas allowed us to determine the proportional percentage amount of each component. The identification of compounds using GC–MS spectra involved leveraging the National Institute of Standards and Technology (NIST) database, which contains approximately 62,000 patterns. The spectra of the unknown components were compared against both the known components stored in the NIST collection and the database’s extensive collection of patterns. The NIST database, which contains more than 62,000 patterns, was used for the GC–MS/MS experiment. A comparison was made between the spectra of the unknown components and the spectrum of known components kept in the NIST collection. The components of the test materials’ names, molecular weights, and structures were determined and listed in Table 1 (Sermakkani and Thangapandian, 2012).

**TABLE 1:** A total number of 39 compounds identified by GC–MS/MS analysis from the leaf extract of *C. ovalis* with their molecular weight and PubChem ID.

Serial	Retention time	% concentration	Compound	Molecular weight	Formula	PubChem ID
1	3.526	12.59	Carvacrol, TBDMS derivative	264.48	C_16_H_28_OSi	13581204
2	3.7	4.50	3,3-Dimethoxy-2-butanone	132.16	C_6_H_12_O_3_	140871
3	3.815	5.29	3,5-Octadiynedioic acid, dimethyl ester	398.5	C_16_H_14_O_4_S_4_	11502178
4	3.895	2.86	3-Methylbenzyl alcohol, TBDMS derivative	384.7	C_17_H_32_O_4_Si_3_	22967275
5	4.18	2.46	Cyclopentene, 1-ethenyl-3-methylene-	68.12	C_5_H_8_	8882
6	4.369	1.25	p-Xylene	106.16	C_8_H_10_	7809
7	4.379	2.82	Dibenzo[b,f][1,4]diazocine, 5,6,11,12-tetrahydro-5-methyl-	206.24	C_14_H_10_N_2_	11969075
8	8.655	0.46	2,6-Dihydroxyacetophenone, 2TMS derivative	296.51	C_14_H_24_O_3_Si_2_	91740707
9	10.817	1.82	Phenol, 3,5-bis(1,1-dimethylethyl)-	206.32	C_14_H_22_O	70825
10	10.901	2.28	3-Hydroxymandelic acid, 3TMS derivative	384.7	C_17_H_32_O_4_Si_3_	522304
11	14.232	1.12	2,6-Dihydroxybenzoic acid, 3TMS derivative	370.66	C_16_H_30_O_4_Si_3_	520869
12	15.661	56.75	Hexadecanoic acid, methyl ester	270.5	C_17_H_34_O_2_	8181
13	15.885	1.55	10-Hydroxydecanoic acid, methyl ester	202.29	C_11_H_22_O_3_	520259
14	15.9	1.99	Tetradecanoic acid, 12-methyl-, methyl ester	256.42	C_16_H_32_O_2_	21206
15	15.94	1.99	Tridecanoic acid, 4,8,12-trimethyl-, methyl ester	270.5	C_17_H_34_O_2_	560155
16	17.68	0.42	Cyclooctasiloxane, hexadecamethyl-	593.2	C_16_H_48_O_8_Si_8_	11170
17	18.377	9.30	9-Octadecenoic acid, methyl ester, (E)-	296.5	C_19_H_36_O_2_	5280590
18	18.806	29.78	Methyl stearate	298.5	C_19_H_38_O_2_	8201
19	18.96	5.58	Heneicosanoic acid, methyl ester	340.6	C_22_H_44_O_2_	22434
20	19.06	2.44	Methyl 9-methyltetradecanoate	256.42	C_16_H_32_O_2_	554137
21	19.105	0.94	Tetradecanoic acid, 5,9,13-trimethyl-, methyl ester	284.5	C_18_H_36_O_2_	554056
22	21.613	1.35	Heneicosane	296.6	C_21_H_44_	12403
23	23.241	1.59	Eicosane	282.5	C_20_H_42_	8222
24	24.829	3.96	2-Methylhexacosane	380.7	C_27_H_56_	150931
25	24.903	5.37	9-Hexadecenoic acid, methyl ester, (Z)-	268.4	C_17_H_32_O_2_	643801
26	29.28	0.44	3,7-Dimethyl-6-nonen-1-ol	212.33	C_13_H_24_O_2_	5363308
27	30.859	0.63	Distearin	625	C_39_H_76_O_5_	102615
28	31.23	0.28	2-Naphthalenemethanol, 1-(dimethylamino)-1,2,3,4,4a,5,6,8a-octahydro-.alpha.,.alpha.,4a,8a-tetramethyl-	265.4	C_17_H_31_NO	558550
29	31.343	0.28	4,5-Dichloro-2-nitrobenzoic acid	236.01	C_7_H_3_Cl_2_NO_4_	583642
30	32.13	0.53	Trispiro[4.2.4.2.4.2.]heneicosane	288.5	C_21_H_36_	566316
31	32.505	0.35	Methyl 9-heptadecenoate or 9–17:1	282.5	C_18_H_34_O_2_	10902087
32	32.95	1.30	3-Methylsalicylic acid, 2TMS derivative	296.51	C_14_H_24_O_3_Si_2_	624536
33	33.575	0.38	Traumatic acid, (E)-, 2TMS derivative	372.6	C_18_H_36_O_4_Si_2_	5463675
34	34.44	1.03	Dihydroartemisinin, 10-O-(t-butyloxy)-	356.5	C_19_H_32_O_6_	537898

### 2.4 *In vitro* test

#### 2.4.1 *In vitro* thrombolytic activity

Streptokinase (SK) was used as the reference standard for the evaluation of the thrombolytic activity of crude methanolic extract (COCME), petroleum ether soluble fraction (COPESF), dichloromethane soluble fraction (CODMSF), ethyl acetate soluble fraction (COEASF), and aqueous-soluble fraction (COASF) of the leaf extract of *C. ovalis* using the technique developed by Prasad et al. (2006).

At first, six Eppendorf tubes were weighed, and then, 500 μL of the volunteers’ venous blood was placed in each tube and incubated for 45 min at 37°C. After the coagulation formed, the serum was removed from this tube. To confirm the weight of the clot, each tube was reweighed. Then, 100 μL of aforementioned fractions and standard streptokinase were correctly added to each Eppendorf tube. Each tube underwent a further 90-min incubation at 37°C for coagulation. After incubation, the fluid was removed, and the tubes were weighed again to measure the weight change following clot disruption. The final percentage of clot lysis was determined by measuring the weight difference before and after the clot lysis process.

The ultimate percentage of clot lysis was calculated using the weight difference between before and after clot lysis.

$$\%\text{ clot lysis}=\frac{Weight\;of\;the\;lysis\;clot}{Weight\;of\;the\;clot\;before\;lysis}\times 100\%.$$

#### 2.4.2 *In vitro* membrane-stabilizing activity

The approach published in the work of Sikder et al. (2012) was used to assess the membrane-stabilizing ability of the plant samples. This method involves hemolysis brought on by heat and hypotonic solution. The blood was collected, and ethylenediaminetetraacetic acid was used as an anticoagulant. Then, the blood was washed with an isotonic solution (0.9% NaCl) and centrifuged for 10 min at 3,000 rpm speed. This washing was carried out three times, and finally, the stock erythrocyte was collected.

##### 2.4.2.1 Hemolysis induced by a hypotonic solution

A hypotonic solution was used during the experimentation. The test material, including COCME, COPESF, CODMSF, COEASF, and COASF at a dose of 2.0 mg/mL and aspirin (standard) at a dose of 0.10 mg/mL, was mixed separately with 0.5 mL stock erythrocyte suspension, 4.5 mL hypotonic solution (0.3% NaCl), and 10 mM sodium phosphate buffer (pH 7.4). For the negative control group, water is used in place of the test material. The mixes underwent a 10-min incubation at room temperature, followed by a 10-min centrifugation at 3,000 rpm.

Finally, the supernatant’s absorbance [optical density (OD)] was taken at 540 nm using a UV spectrophotometer.

The hemolysis percentage was calculated using the following equation:

$$\%\text{ hemolysis}=1-\frac{OD2}{OD1}\times 100\%.$$

Here, OD1 is the optical density of the hypotonic-buffered saline solution alone (control group); OD2 is the optical density of the test sample in the hypotonic solution.

##### 2.4.2.2 Hemolysis induced by heat

At first, 4.5 mL of isotonic solution and 10 mM sodium phosphate buffer solution and the standard aspirin at a dose of 0.10 mg/mL or the abovementioned samples at a dose of 1 mg/mL were poured into two sets of different centrifugal tubes. Then, 30 µL of stock erythrocytes were mixed with each of the tubes. One set of tubes was incubated in a water bath at 54°C for 20 min. The second pair was kept in an ice bath at 0°C–50°C for the same time. After that, these mixtures were centrifuged for 3 min at 3,000 rpm speed, and then, UV spectrophotometric absorbance was taken at 540 nm.

The hemolysis percentage was calculated using the following equation:

$$\%\text{ hemolysis}=1-\frac{OD2-OD1}{OD3-OD1}\times 100\%.$$

Here, OD 1 is the test sample unheated, OD 2 is test sample heated, and OD 3 is the control sample heated.

### 2.5 *In vivo* test

#### 2.5.1 Test animal

Swiss albino mice aged 4–5 weeks of either sex were procured from the International Center for Diarrheal Disease and Research in Bangladesh for use in *in vivo* biological experiments (ICDDR, B). They received free access to rodent chow and water while being housed at a constant room temperature of 24°C ± 2°C and relative humidity levels of 60%–70% for 12 h of light and darkness. However, food was withheld 12 h prior to the experiment and throughout its duration. The care and use of experimental animal guidance were followed in conducting the studies. The institution’s ethics committee gave its approval to the procedures for using animals in the tests.

#### 2.5.2 Study design for the *in vivo* test

The experimental models consisted of five groups of Swiss albino mice, with each group containing four mice, and the assignment to the groups was performed randomly. For the various experimental models, five groups of three Swiss albino mice were randomly assigned, where each group contained four mice. The negative control group, serving as the first group, received 10 mL/kg of distilled water. The first group acted as the negative control and received 10 mL/kg of distilled water. The second group acted as the experiment’s positive control and was administered normal medications. The third, fourth, and fifth groups received 200, 400, and 600 mg/kg b.w dose of COCME, respectively.

#### 2.5.3 *In vivo* central analgesic activity

The tail immersion method described by Ezeja et al. (2011) was used to determine the central analgesic activity of COCME. Diclofenac sodium (50 mg/kg bw) solution was administrated as standard. COCME (200, 400, and 600 mg/kg bw) for test groups and distilled water for the negative control group were administrated orally. In order to conduct the test, 1–2 cm of the mouse’s tail was dipped into a water bath filled with warm water that was kept at a constant temperature of 55°C. The mouse’s tail deflection time was timed at 0, 30, 60, and 90 min after the treatments were administered.

The following equation was used to calculate the % time elongation, which was then compared to the reference value to assess the central analgesic action.

The central analgesic action of the group increases with group elongation %.

$$\%\text{ time elongation}=\frac{T\;test-T\;control}{T\;contrl}\times 100\%.$$

Here, *Ttest* is the average time of tail deflection in the test group, and *Tcontrol* is the average time of tail deflection in the control group.

#### 2.5.4 *In vivo* peripheral analgesic activity

To ascertain the peripheral analgesic effect of *C. ovalis* in albino mice, the acetic acid-induced writhing test was carried out as previously reported (Satyanarayana et al., 2004). Diclofenac sodium (50 mg/kg bw) solution was injected intraperitoneally as standard. Test groups were given three different doses including 200, 400, and 600 mg/kg bw of COCME, and the negative control group was given only distilled water. After 30 min of sample administration, glacial acetic acid (0.1 mL/30 mg bw) was injected through the intraperitoneal route. For each group of mice, the number of abdominal constrictions, or writhes, was measured from 5 min after the injection of acetic acid until 10 min later, and the percentage inhibition of writhing was calculated using the following formula to assess the peripheral analgesic activity:

$$\%\text{ inhibition of writhing}=\frac{W\;test-W\;control}{W\;contrl}\times 100\%.$$

Here, *Wtest* refers to the average number of writhing in the test group, and *Wcontrol* refers to that in the control group.

### 2.6 *In silico* study

#### 2.6.1 Docking software

The molecules were subjected to computational docking analysis, employing widely recognized software applications such as PyRx, PyMOL 2.3, Discovery Studio 4.5, and Swiss-PdbViewer, to explore their molecular interactions and binding affinities in a virtual environment (Hasnat et al., 2023).

#### 2.6.2 Molecular docking: ligand preparation

The structures of 39 isolated compounds of the *C. ovalis* leaf extract and aspirin (PubChem CID- 2,244), diclofenac sodium (PubChem CID- 3,033), and streptokinase (PubChem CID- 9815560) were downloaded from the PubChem database and are presented in Table 1 (https://pubchem.ncbi.nlm.nih.gov/). In order to obtain the best possible hit rate against the aforementioned targets, the ligands were downloaded in the 3DSDF format. These ligands, along with their PubChem CIDs, were serially loaded in the Discovery Studio 4.5. It should be mentioned that the Pm6 semiempirical technique was used to optimize all phytochemicals in order to improve docking accuracy (Bikadi and Hazai, 2009).

#### 2.6.3 Molecular docking: protein preparation

Three-dimensional crystal structures, including the mu-opioid receptor (PDB ID: 5C1M) for central analgesic activity (Ahmed *et al.*, n.d.), cyclooxygenase-2 (COX-2) [PDB ID: 1CX2] for peripheral analgesic activity (Muhammad et al., 2015), tumor necrosis factor alpha (TNF-α) [PDB ID: 2AZ5] for membrane-stabilizing activity (Kumar et al., 2021), and tissue plasminogen activator (TPA) [PDB ID: 1A5H] for thrombolytic activity (Emon *et al.*, n.d.), were collected from the RCBS Protein Data Bank (https://www.rcsb.org/structure) in the PDB format. Using Discovery Studio 2020, all of the water and the heteroatoms were taken out of the proteins. Then, using the Swiss-PdbViewer’s energy reduction software, all the biomolecules were organized by adding nonpolar hydrogen atoms and kept in their lowest energy state for future research.

#### 2.6.4 Ligand–protein interaction

To forecast potential binding profiles of isolated compounds with their binding affinities to the target molecules, the present computer-aided ligand–protein interaction was drawn (Bikadi and Hazai, 2009). Regarding the process of linking molecular drugs to proteins, for this molecular drug–protein linking procedure, highly sophisticated PyRxAutodock Vina was used, and semi-flexible modeling was used for the molecular docking. Initially, the protein was loaded and prepared to interact with the target macromolecule. To ensure specific ligand binding to the target, we selected amino acids with their respective IDs from the literature. The protein was first loaded and formatted to the target macromolecule, and the literature-based amino acids with their ID were chosen to ensure target-specific ligand binding.

For the 5C1M target, Asp 147, Tyr 148, Met 151, Lys 233, Trp 293, Ile 296, His 297, Val 300, Ile 322, Gly 325, and Tyr 326 amino acids from A chain were selected for site-targeted docking (Ahmed et al., 2021). However, His 90, Gln 192, Val 349, Leu 352, Ser 353, Tyr 355, Tyr 385, Ala 516, Phe 518, Val 523, Ala 527, and Ser 530 amino acids of 1CX2 were selected (Muhammad et al., 2015). As standard, diclofenac sodium was used to check its affinity with these receptors. Additionally, TYR 59, TYR 119, LEU 120, GLY 121, and TYR 151 amino acids of the A chain and TYR 59, SER60, GLN 61, TYR 119, LEU 120, and GLY 121 amino acids of the B chain of 2AZ5 were picked for site-specific docking (Kumar et al., 2021). Here, aspirin was used as standard. Using PrankWeb, Arg 39, Leu 41, Cys 42, His 57, Cys 58, Gln 60, Glu 60A, Phe 60C, Tyr 99, Tyr 151, Asp 189, Ala 190, Cys 191, Gln 192, Gly 193, Ser 195, Ile 213, Ser 214, Trp 215, Gly 216, Leu 217, Gly 219, Val 224, Pro 225, Gly 226, Val 227, and Tyr 228 amino acids of 1A5H were chosen for site-specific docking (Jendele et al., 2019). Streptokinase was employed as a reference and as a thrombolytic drug to determine the binding affinity with 1A5H and compare it to the identified compounds’ affinity toward the receptor.

Next, in order to find the most optimal binding interactions with the selected macromolecules, all the ligands’ PDB files were imported and then converted to the pdbqt format using the Open Babel tool within the PyRxAutoDock Vina software. Subsequently, the minimized ligand structures were employed for docking simulations.

Second, to match the best optimal hit during the docking against these selected macromolecules, all the PDB files of the ligands were imported and afterward minimized into the pdbqt format with the Open Babel tool in the PyRxAutoDock Vina software.

Finally, the grid box was created by maintaining the protein’s active binding sites inside the box’s center, which was designated as the grid mapping center. For the mu receptor center, X = −0.8683, Y = 14.0586, and Z = −57.3660, with dimensions X = 19.0100, Y = 18.1292, and Z = 19.8212, were maintained for the grid box. For the COX-2 grid mapping center, X = 22.5615, Y = 21.2666, and Z = 15.7556, with dimensions X = 22.59057, Y = 17.8973, and Z = 24.1880. However, for the TNF-alpha receptor grid mapping center, X = −19.8828, Y = 73.8134, and Z = 37.9172, with dimensions X = 19.7402, Y = 22.1537, and Z = 13.7094. Finally, for the 1A5H center, X = 5.5242, Y = 35.06182, and Z = 49.2687, with dimensions X = 26.8695, Y = 23.9366, and Z = 30.8053 for the grid box, were maintained.

Upon docking, the remaining parameters were set to their default values. Subsequently, using AutoDock Vina (version 1.1.2), computer-based-aided molecular docking of the ligand compounds was carried out while maintaining all essential conditions default (Ahmed et al., 2021). Finally, BIOVIA Discovery Studio version 4.5 was used to conceptualize all docking analyses for extrapolating and concluding the best-fitted figures using 2D and 3D configurations.

#### 2.6.5 ADME/T study

pkCSM (http://structure.bioc.cam.ac.uk/Pkcsm) was used to evaluate the absorption, distribution, metabolism, excretion, and toxicological (ADME/T) parameters (Pires et al., 2015). Meanwhile, Swiss ADME (http://www.swissadme.ch) was used to determine the drug-likeliness and bioavailability score of selected compounds (Daina et al., 2017).

### 2.7 Statistical analysis

The data were presented as a standard error mean (SEM). Statistical analyses using one-way ANOVA were conducted followed by Dunnett’s multiple comparison tests. The observed values were compared to the control group and were considered statistically significant at *p* < 0.05, *p* < 0.01, and *p* < 0.001 (Miah et al., 2018).

## 3 Results

### 3.1 GC–MS/MS analysis

**FIGURE 1:**
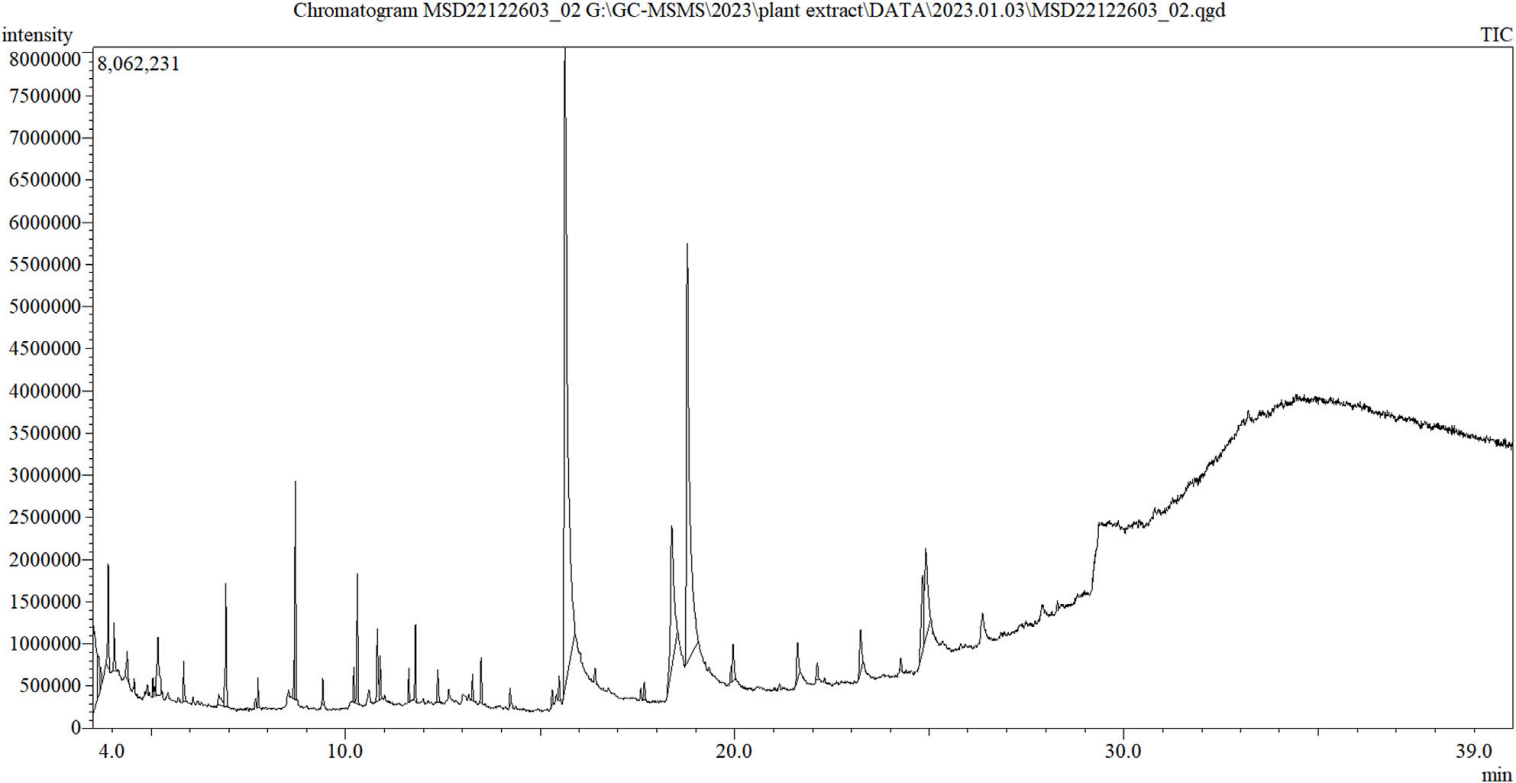
GC–MS/MS chromatograph of the crude methanolic extract of leaves of *C. ovalis*.

The sample produced 39 peaks on the GC–MS/MS chromatogram (Figure 1), indicating 39 different phytochemical components. Following a comparison of the mass spectra of the constituents in the NIST library with those of the picks, 39 phytocompounds were defined and identified (Table 1).

The maximum peak area was found for hexadecanoic acid [methyl ester] (56.749%), methyl stearate (29.782%), carvacrol [TBDMS derivative] (12.586%), 9-octadecenoic acid [methyl ester, (E)-] (9.297%), heneicosanoic acid [methyl ester] (5.580%), 9-hexadecenoic acid [methyl ester, (Z)-] (5.372%), 3,5-octadiynedioic acid [dimethyl ester] (5.291%), 3,3-dimethoxy-2-butanone (4.496%), 2-methylhexacosane (3.963%), 3-methylbenzyl alcohol, TBDMS derivative (2.856%), dibenzo[b,f][1,4]diazocine, 5,6,11,12-tetrahydro-5-methyl- (2.817%), cyclopentene, 1-ethenyl-3-methylene- (2.456%), methyl 9-methyltetradecanoate (2.438%), 3-hydroxymandelic acid [3TMS derivative] (2.275%), [1,3,5]triazine-2-carboxylic acid, 4-ethylamino-6-morpholin-4-yl-, amide (2.131%), etc. The remaining compounds have a peak area of less than 2%.

### 3.2 Thrombolytic activity

**FIGURE 2:**
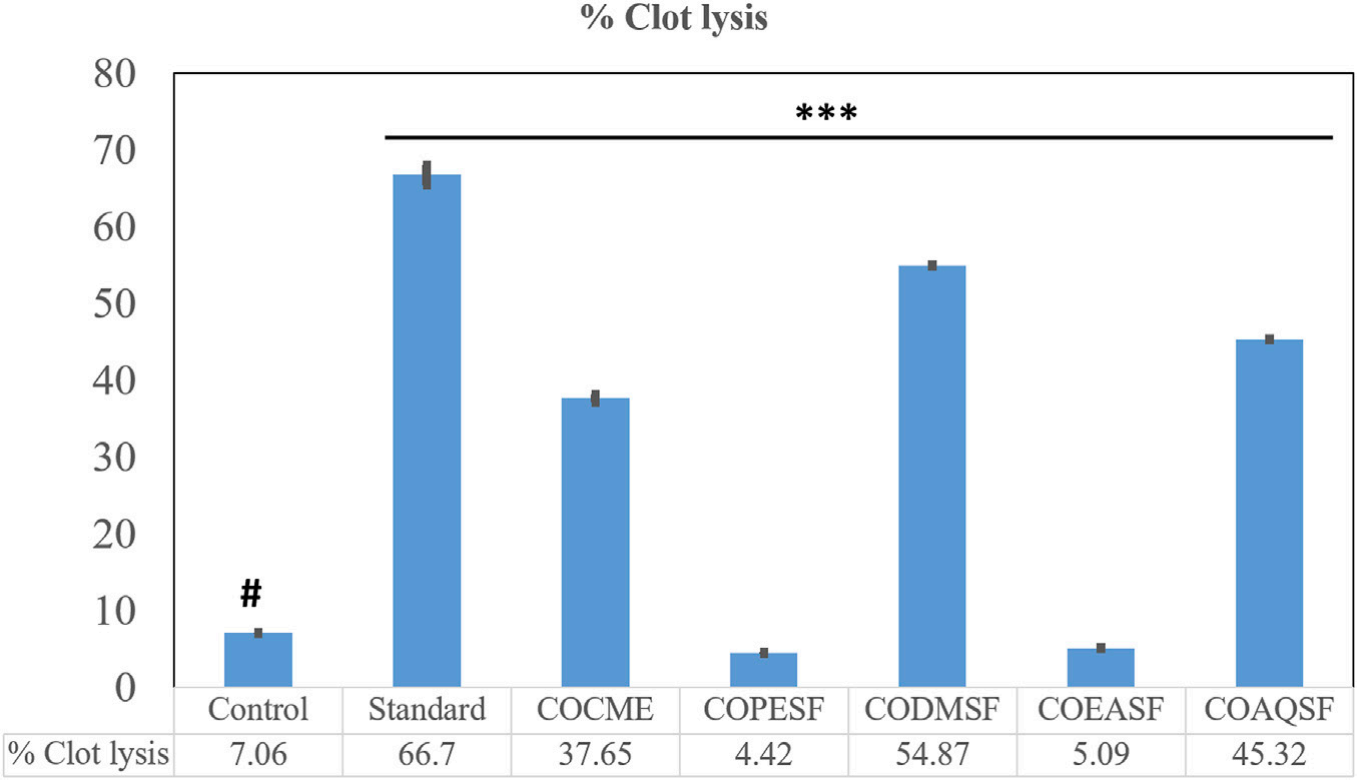
Thrombolytic effect of different fractions of the *C. ovalis* leaf extract. Each value is expressed as mean percentage (n = 3), *p < 0.05, **p < 0.01, and ***p < 0.001, and compared with the control group.

In this investigation, the CODMSF exhibited an extremely strong thrombolytic impact by inhibiting 54.87% of the clot, which is close to the standard streptokinase (66.66%) (Figure 2). Additionally, COCME and COAQSF fractions demonstrated noteworthy anti-thrombosis effects through suppressing clots by 37.65% and 45.32%, respectively, while the control group only showed 7.06% clot lysis. This result signifies that there is a huge opportunity to develop new thrombolytic drugs from *C. ovalis*.

### 3.3 Membrane-stabilizing activity

**FIGURE 3:**
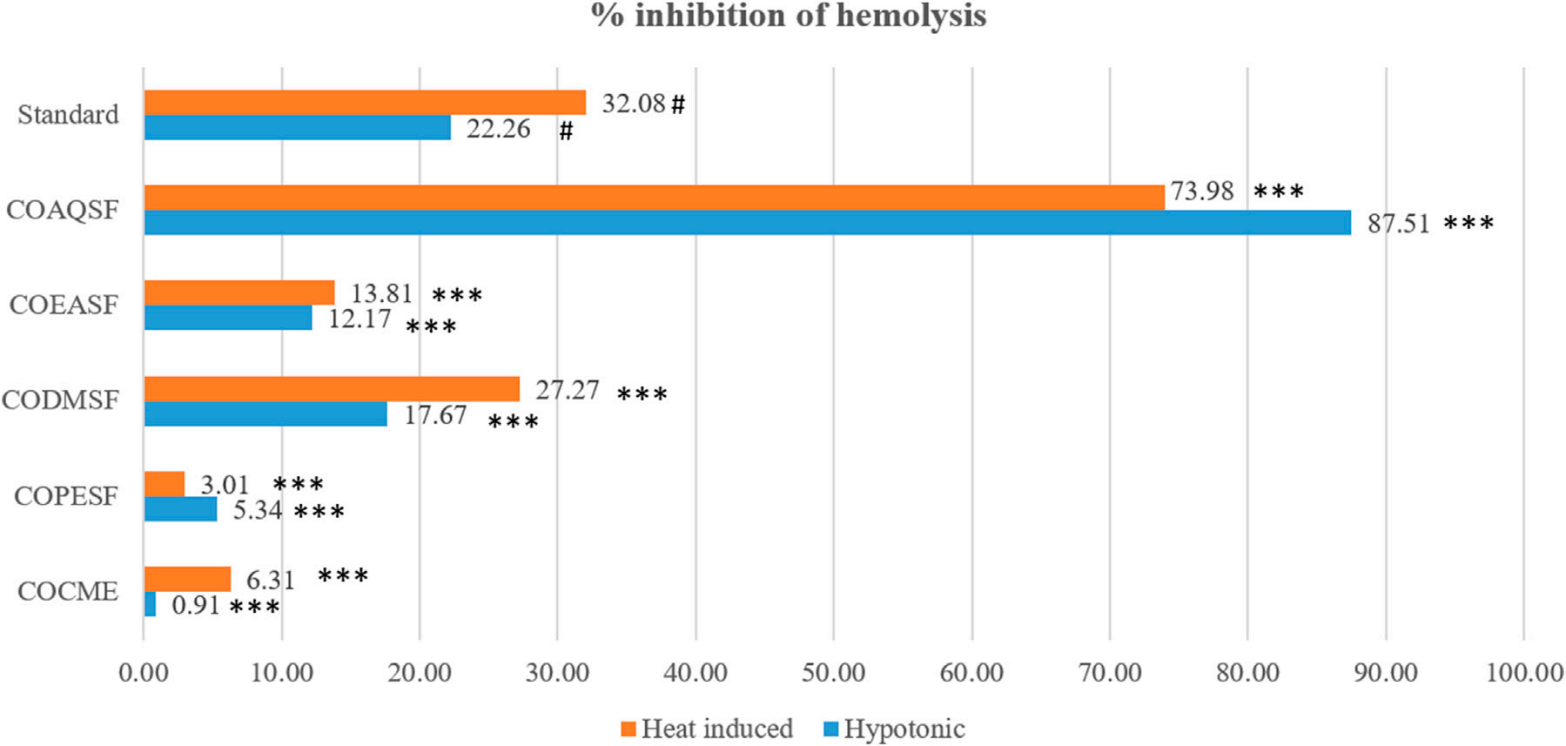
Membrane-stabilizing effect of different fractions of the *C. ovalis* leaf extract. Each value is expressed as mean percentage (n = 3), *p < 0.05, **p < 0.01, and ***p < 0.001, and compared with the standard group.

Our study demonstrated that the COAQSF has manifested remarkable membrane-stabilizing activities exceeding aspirin (standard) activities with twice its value. Figure 3 illustrates that COAQSF inhibited 73.98% and 87.51% hemolysis against heat and hypotonic solution-induced hemolysis, respectively, while the values were 3.08% and 22.26%, respectively, for the standard streptokinase. However, CODMSF exhibited prominent activity followed by COEASF, where the inhibition of hemolysis was measured at 27.27% and 17.67% for COSMSF and 13.81% and 12.17% for COEASF against heat- and hypotonic solution-induced hemolysis, respectively. This finding enhanced the probability of developing new remedies against inflammation.

### 3.4 Central analgesic activity

By extending the experimental mice’s tail immersion period, the various dosages of the crude methanolic extract of *C. ovalis* (200, 400, and 600 mg/kg) demonstrated considerable central analgesic efficacy. A dose- and time-dependent increase in the activity was observed. The highest activity was observed for 600 mg/kg extract which increased the tail emerged time to approximately 139.75 ± 0.23, 222.34 ± 0.07, and 381.14% ± 0.55%, respectively, after 30, 60, and 90 min of dose ingestion (Table 2).

**TABLE 2:** Central analgesic effect of different doses of the crude methanolic extract of the leaves of *C. ovalis* in the mice model. Each value is expressed as mean ± SEM (n = 4). *p < 0.05, **p < 0.01, ***p < 0.001 and compared with the control group (only distilled water).

Treatment	% time elongation (mean ± SEM)		
	At 30 min	At 60 min	At 90 min
Standard	146.75 ± 0.16	345.66 ± 0.25^**^	683.12 ± 0.34^**^
Control	35.67 ± 0.11	24.92 ± 0.64	24.30 ± 0.91
COCME 200	45.34 ± 0.26^*^	102.92 ± 0.22^**^	203.14 ± 0.15^**^
COCME 400	132.58 ± 0.23^**^	193.92 ± 0.16^**^	309.37 ± 0.21^**^
COCME 600	139.75 ± 0.23^**^	222.34 ± 0.07^**^	381.14 ± 0.55^**^

### 3.5 Peripheral analgesic activity

A remarkable peripheral analgesic activity was observed for different dosages of the COCME, especially for the dose of 400 mg/kg and 600 mg/kg. In comparison to the control (34.5 mean writhings), an average of 16.5 mean writhings was witnessed in the 200 mg/kg dose group, and 9.5 mean writhings were noted in the 400 and 600 mg/kg extract groups (Table 3). This indicates that the 200, 400, and 600 mg/kg COCMEs reduced writhing by approximately 32.65 ± 1.19, 61.22 ± 1.04, and 61.22% ± 1.85% , respectively. All of the data were significantly justified.

**TABLE 3:** Peripheral analgesic effect of different doses of the crude methanolic extract of the leaves of *C. ovalis* in the mice model. Each value is expressed as mean ± SEM (n = 4), *p < 0.05, **p < 0.01, and ***p < 0.001, and compared with the control group (only distilled water).

Test groups	Mean writhing	% inhibition of writhing (mean ± SEM)
Control	24.5	
Standard	4	83.67 ± 0.41^**^
COCME 200	16.5	32.65 ± 1.19^*^
COCME 400	9.5	61.22 ± 1.04^**^
COCME 600	9.5	61.22 ± 1.85^*^

### 3.6 Molecular docking study

**FIGURE 4:**
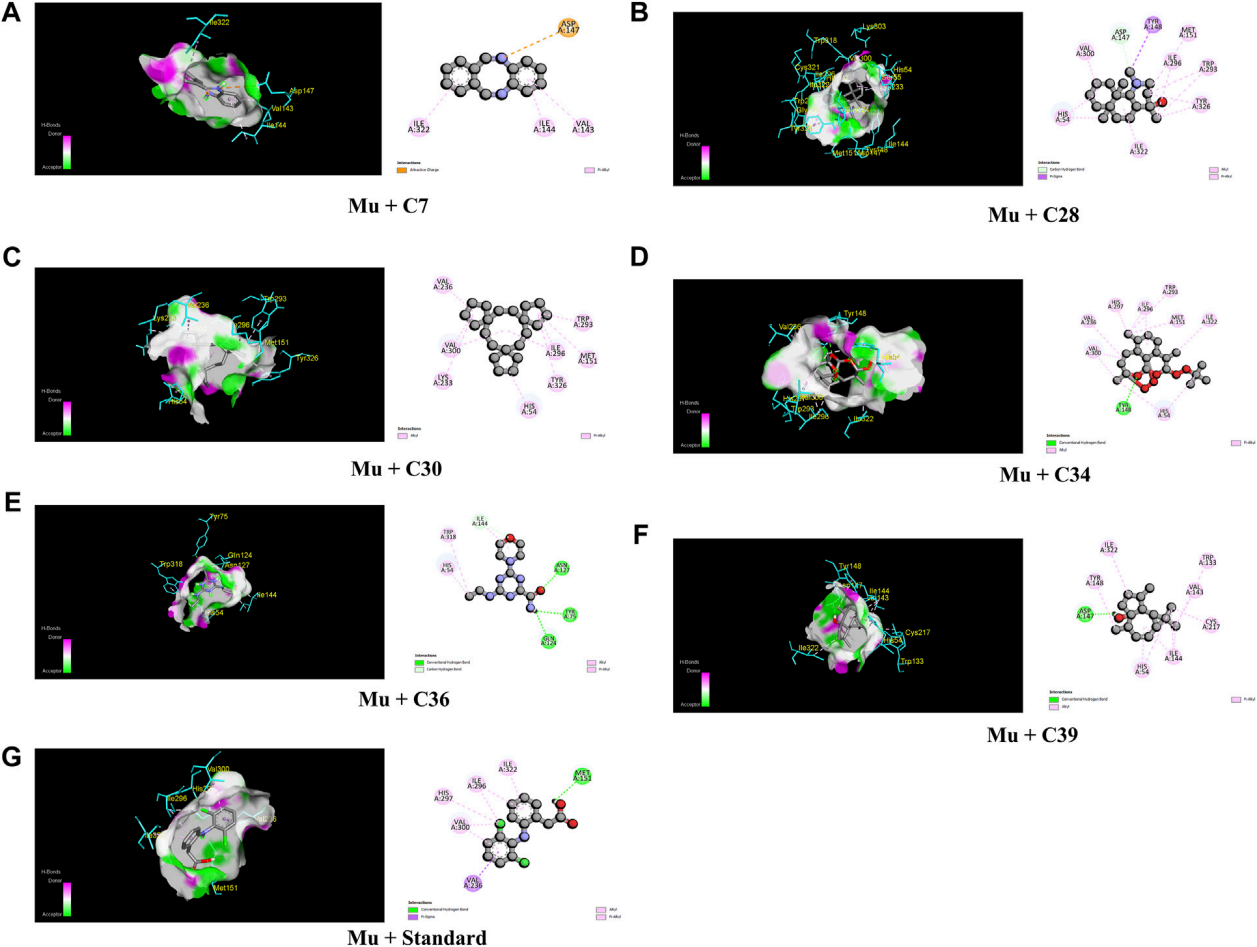
The best critical interactions in the binding pocket for the chosen ligands and receptors are represented in 3D and 2D, where A to G represent the interaction of C7, C28, C30, C34, C36, C39, and standard (diclofenac sodium) with the mu-opioid receptor, respectively.

The lowermost activity was observed by C30 with a binding affinity of −8.8 kcal/mol followed by C34, C7, C36, C28, and C39, which exhibited binding affinities of −8.5, −8.1, −7.7, −7.3, and −7.2 kcal/mol, respectively, against the Mu receptor compared to the standard diclofenac sodium, which scored −7.6 kcal/mol (Table 4). Figure 4 shows that C30 attached to eight amino acids of Mu receptors, namely, HIS 54, MET 151, LYS 233, VAL 236, TRP 293, ILE 296, VAL 300, and TYR 326, while C34 attached to nine amino acids, namely, HIS 54, TYR 148, MET 151, VAL 236, TRP 293, ILE 296, HIS 297, and VAL 300. In comparison, the standard diclofenac is bound through six amino acids, namely, MET 151, ILE 296, HIS 297, ILE 322, VAL 300, and VAL 236.

**TABLE 4:** Binding affinities of the identified compounds and standards against four receptors representing central and peripheral analgesic, membrane-stabilizing, and thrombolytic activities.

Compound	Binding affinity			
	Central analgesic	Peripheral analgesic	Membrane stabilizing	Thrombolytic
	Mu	COX-2	TNF-α	TPA
C1	−3	−3.1	−3	−3.3
C2	−4	−4.4	−4.2	−4.2
C3	−6.4	−4.9	−6.1	−5.8
C4	−3.1	−3.1	−3	−3.3
C5	−3.5	−3.8	−3.5	−4.1
C6	−5	−5.4	−4.7	−6.1
C7	−8.1	−9	−7.5	−7.4
C8	−1.2	−1.1	−0.9	−1.1
C9	−6.6	−6.6	−6.5	−5.2
C10	−1.2	−1	−0.9	−1.1
C11	−1.2	−1	−0.9	−1.1
C12	−5.4	−6.3	−5.4	−5.5
C13	−5.1	−5.7	−4.7	−5.2
C14	−5.6	−6.3	−5.1	−5.4
C15	−6.1	−7.1	−5.6	−6
C16	−1.5	−1.5	−1.1	−1.6
C17	−6	−6.7	−5.4	−5.5
C18	−5.9	−6.8	−5.1	−5.7
C19	−5.7	−6.9	−5.4	−5.3
C20	−5.8	−6.3	−5.5	−5.8
C21	−6.1	−7.2	−5.8	−5.7
C22	−6	−7	−5.4	−5.4
C23	−5.3	−6.7	−4.9	−5.6
C24	−6.4	−6.6	−5.2	−5.5
C25	−5.5	−6.5	−5	−5.8
C26	−5.8	−6.4	−5.8	−5.9
C27	−6.9	−9.5	−7.5	−6.9
C28	−7.3	−5.5	−7.5	−5.7
C29	−6.1	−6.5	−6.4	−6.1
C30	−8.8	−6.3	−9.3	−7.4
C31	−5.8	−6.7	−5.2	−5.3
C32	−1.2	−1.1	−0.9	−1.1
C33	−1.2	−1	−0.9	−1.1
C34	−8.5	−6.5	−7.9	−6.7
C35	−6.3	−6.3	−5.3	−5.6
C36	−7.7	−7.8	−6.5	−6.9
C37	−1.2	−1	−0.9	−1.1
C38	−1.2	−1	−0.9	−1.1
C39	−7.2	−8.4	−7.5	−5.9
Aspirin	-	-	−5.8	-
Diclofenac sodium	−7.6	−7.9	-	-
Streptokinase	-	-	-	−6.3

**FIGURE 5:**
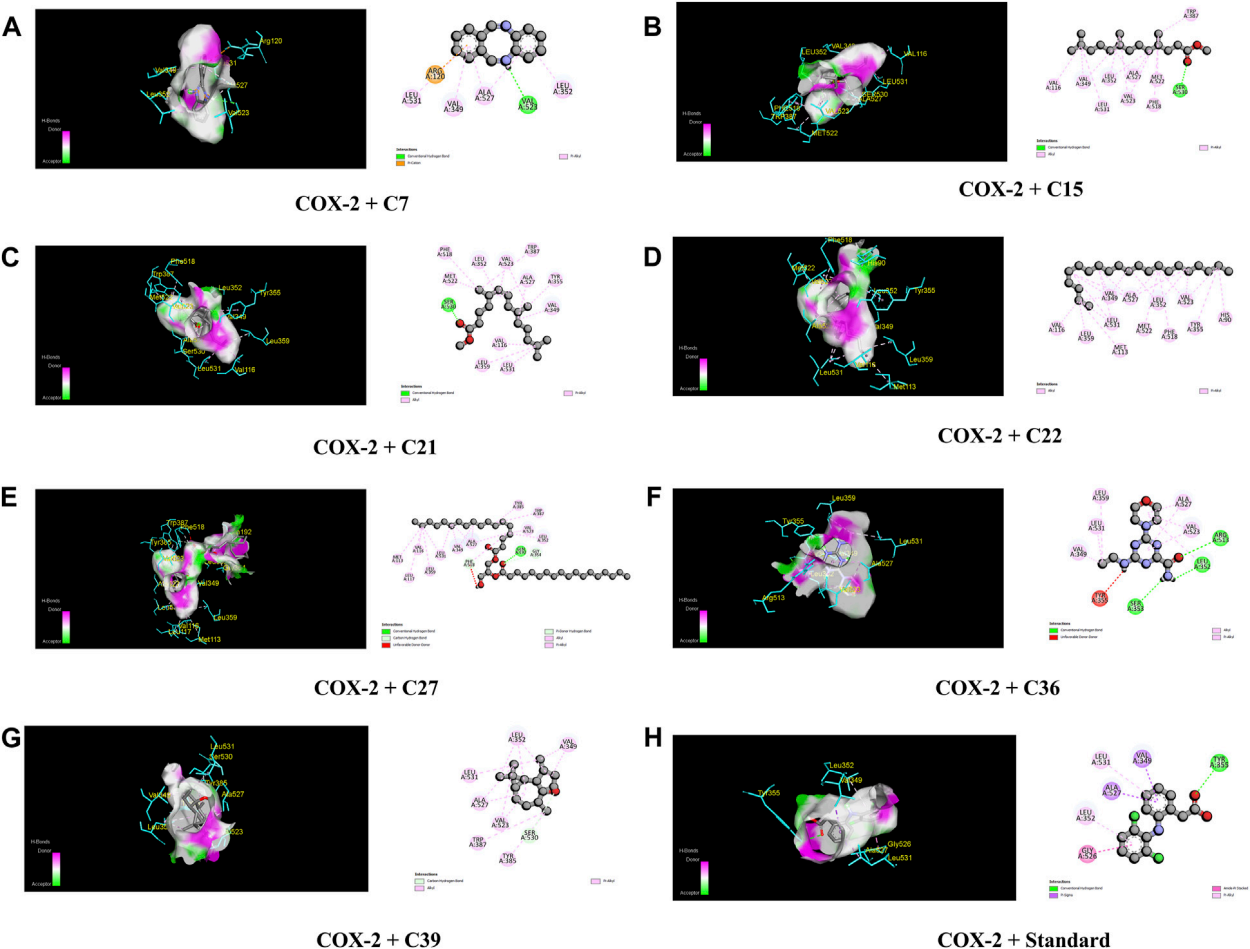
The best critical interactions in the binding pocket for the chosen ligands and receptors are represented in 3D and 2D, where A to H represent the interaction of C7, C15, C21, C22, C27, C36, C39, and standard (diclofenac sodium) with the COX-2 receptor, respectively.

Table 4; Figure 5 illustrate that C27 exhibited the highest efficacy against the COX-2 receptor through a series of bonds with the amino acids including MET 113, VAL 116, LEU 117, GLN 192, VAL 349, LEU 352, GLY 354, LEU 359, TYR 385, TRP 387, PHE 518, VAL 523, ALA 527, and LEU 531 with a binding score of −9.5 kcal/mol. However, C7 is bound to six amino acids of COX-2, namely, ARG 120, LEU 531, VAL 349, LEU 352, VAL 523, and ALA 527, and shows a binding affinity of −9 kcal/mol. Comparably, the standard diclofenac sodium exhibited a binding affinity of −7.9 kcal/mol and interacted with LEU 531, VAL 349, LEU 352, TYR 355, GLY 526, and ALA 527 of COX-2. Additionally, C39, C36, C21, C15, and C22 showed notable binding affinity of −8.4, −7.8, −7.2, −7.1, and −7 kcal/mol against COX-2, respectively. In addition, approximately 16 compounds exhibited a binding affinity ranging from −6.9 to −6.3 kcal/mol against the receptor.

**FIGURE 6:**
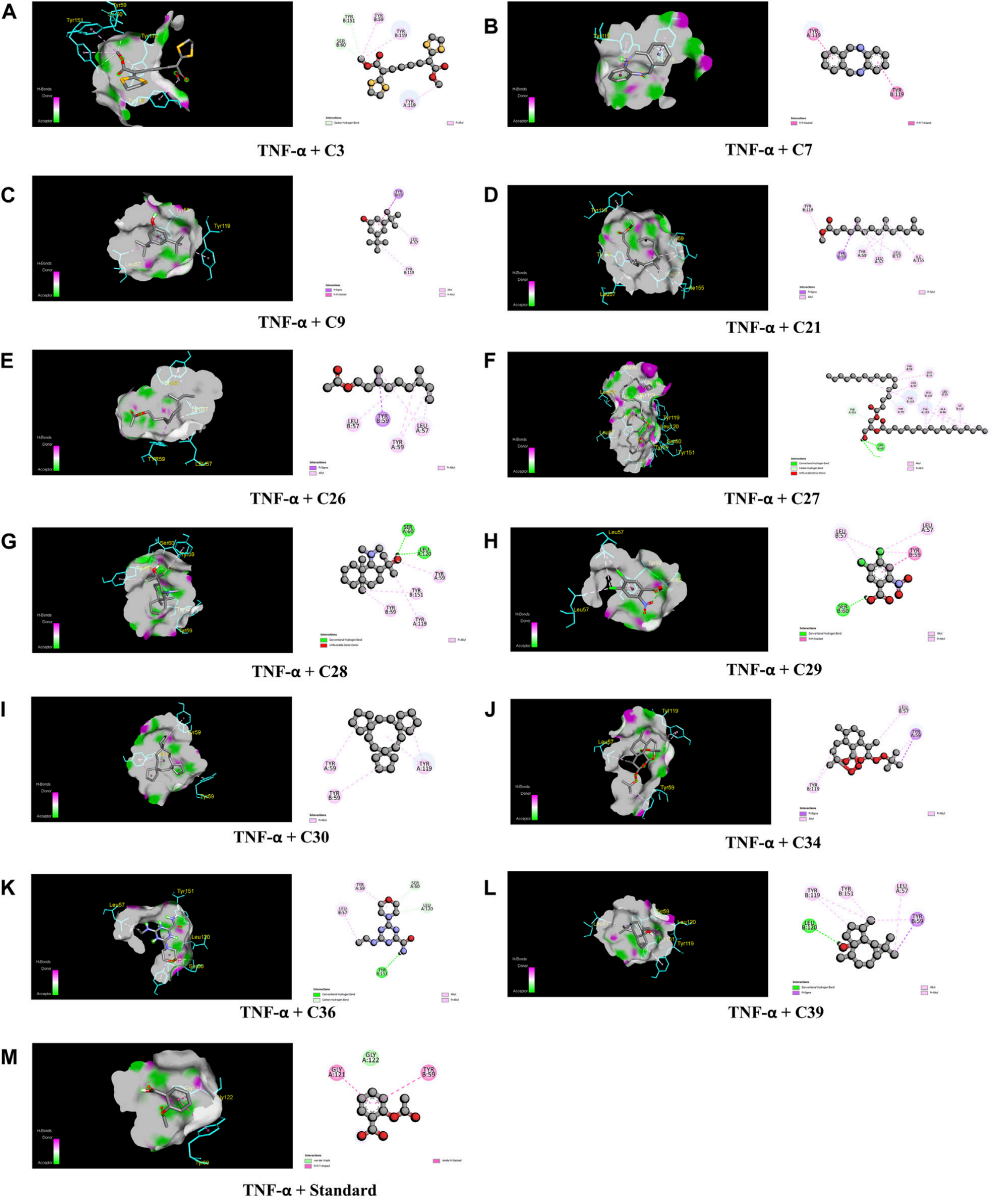
The best critical interactions in the binding pocket for the chosen ligands and receptors are represented in 3D and 2D, where A to M represent the interaction of C3, C7, C9, C21, C26, C27, C28, C29, C30, C34, C36, C39, and standard (aspirin) with the TNF-α receptor, respectively.

When compared to regular aspirin (−5.3 kcal/mol), C30 had a binding affinity for TNF-α (PDB ID: 2AZ5) of −9.3 kcal/mol. C7, C27, C28, and C39 all had binding affinities of −7.5 kcal/mol, whereas C34 had a binding affinity of −7.9 kcal/mol. However, with binding affinities ranging from −6.5 to −5.8 kcal/mol, C3, C9, C21, C26, C29, and C36 scored higher affinities compared to aspirin. In the case of active amino acid residue, C30 was found to bind against TYR 59 TYR 119 of the A chain and TYR 59 of the B chain. Similarly, C39 bound to TYR 59 TYR 119 of the A chain and LEU 57 of the B chain, whereas aspirin bound to HIS 57, TYR 99, ALA 190, CYS 191, GLN 192, GLY 193, TRP 215, and GLY 216 of the A chain (Figure 6).

**FIGURE 7:**
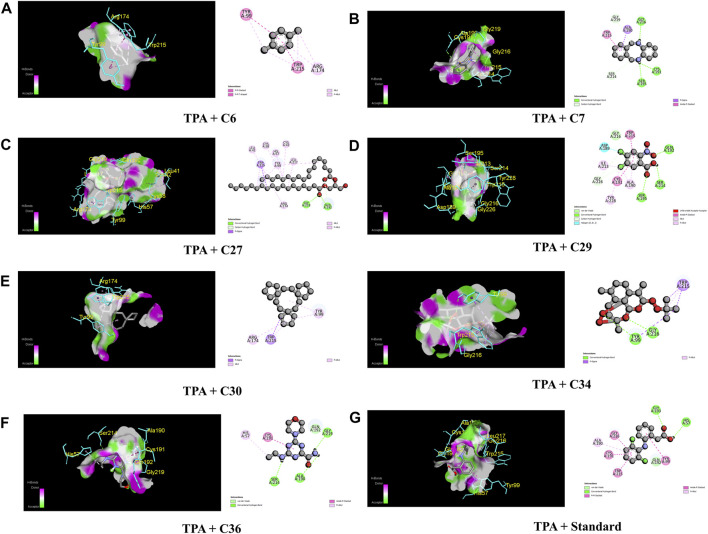
The best critical interactions in the binding pocket for the chosen ligands and receptors are represented in 3D and 2D, where A to G represent the interaction of C6, C7, C27, C29, C30, C34, C36, and standard (streptokinase) with the TPA receptor, respectively.

In the case of TPA (PDB ID: 1A5H), the most prominent affinity was witnessed in C7 and C30 with a binding affinity of −7.4 kcal/mol. On the other hand, C6, C27, C29, C34, and C26 manifested promising affinities with values of −6.1, −6.9, −6.1, −6.7, and −6.9 kcal/mol, respectively, compared to a value of −6.3 kcal/mol of the standard streptokinase. While C30 bound to TYR 99, ARG 174, and TRP 215 amino acids of the TPA receptor in Figure 7, C7 bound to ALA 190, CYS 191, SER 195, SER 214, GLY 216, and GLY 219 amino acids. The active amino acid residues for the streptokinase were HIS 57, TYR 99, ALA 190, CYS 191, GLN 192, GLY 193, TRP 215, and GLY 216, as shown in Figure 7.

## 4 Discussion

The best place to find novel bioactive compounds with new treatments is in medicinal plants. As a result, plant-based natural remedies are widely used in developing nations and given special attention because of their multiple protective benefits and beneficial effects on human health. Approximately 80% of people use traditional medicines, even in underdeveloped nations (Alam et al., 2020a). Oils contained by plant extract are typically intricate blends of vital secondary metabolites that have been extracted from plants, animals, and microorganisms. These mixes contain between 10 and 60 ingredients in varying concentrations, but often just 2–4 main molecules are responsible for the biological features (Rahman et al., 2013). The goal of the current research was to identify the chemical components and assess their biological and pharmacological potential in order to understand the significance of the methanolic crude extract of *C. ovalis*. The study will provide an update, confirm the value of aromatherapy, and add a new bearing on biopotency.

The GC–MS/MS analysis was carried out to identify the various volatile matter components found in the methanolic crude extract of *C. ovalis* leaves, such as alcohols, acids, amides, amines, esters, and hydrocarbons, both in quantitative and qualitative ways. A quantitative measurement of each component’s quantity can be found in peak heights or regions beneath the peaks. This study identified 39 phytocompounds from the methanolic crude extract of leaves of *C. ovalis* while the maximum peak was acquired for hexadecanoic acid [methyl ester], which is approximately 56.749%, indicating the presence of a large quantity of this compound in this extract.

Hexadecanoic acid has been reported for potential antioxidant activity as well as anticancer potential against human colon cancer cells (Bharath et al., 2021). In addition, the compound exhibited significant antibacterial activity against several antibiotic-resistant bacteria including *Staphylococcus aureus* W35, *Pseudomonas aeruginosa* D31, *Klebsiella pneumoniae* DF30, and *K. pneumoniae* B40 (Shaaban et al., 2021). Furthermore, methyl ester of hexadecanoic acid isolated from *Annona muricata* Linn. has been found promising antifungal potential against *Alternaria solani*, *Aspergillus erythrocephalus*, and *Aspergillus albicans* (Abubacker and Deepalakshmi, 2013). However, the presence of a significant amount of hexadecanoic acid in *C. ovalis* leaf extract increases the possibility that it has natural cytotoxic, antibacterial, and antifungal properties.

Our investigation revealed a substantial presence of methyl stearate, constituting approximately 29.782%, known for its medicinal properties. Notably, as a bioactive compound identified in the fermentation broth, methyl stearate plays a pivotal role in inhibiting nematode infection. Its efficacy is demonstrated through actions such as reducing egg hatching, repelling J2s from plant roots, and modulating key parasitic nematode genes (Mi-flp-18 and 16D10). This compound shows promise in effectively controlling *Meloidogyne incognita*. Additionally, its positive influence on enhancing banana plant growth at lower concentrations suggests its potential as an environmentally friendly approach to nematode control. This presents a viable alternative to conventional pesticides, contributing to sustainable agricultural practices (Lu et al., 2020).

Blood flow in the circulatory system is impeded if a thrombus forms inside blood arteries, which can lead to a number of fetal cardiovascular illnesses including hypertension, stroke, and anoxia. Thrombolites are substances that prevent thrombus formation, hence managing and treating embryonic cardiovascular disorders (Islam et al., 2019). Several studies have been conducted in the past using medicinal plants from Bangladesh to identify natural chemicals having anti-thrombolytic potential (Rahman et al., 2013). The research discovered that the dichloromethane (DCM) fraction of the methanolic leaf extract from *C. ovalis* exhibited notable thrombolytic activity. Moreover, both the crude methanolic extract and the aqueous fraction demonstrated encouraging properties in dissolving blood clots, which increases the potential for developing novel preparations derived from this plant to mitigate cardiovascular diseases.

Inflammation causes lysosome lysis, which releases enzymes that cause different types of tissue damage. Inflammation causes lysosome lysis, which releases enzymes that cause different types of tissue damage. When an RBC is exposed to adverse circumstances or substances such as hypotonic medium or heat, the membranes break due to hemolysis and hemoglobin oxidation. In times of enhanced permeability brought on by inflammatory mediators, the stability of the membrane limits the passage of proteins from the blood and fluids into the RBC (Kosala et al., 2018). Thus, by stabilizing RBC induced by both hypotonic solution as well as heat, the aqueous fractions illustrated extremely potent anti-inflammatory activity compared to the standard aspirin. Furthermore, the crude methanolic extract from the leaves of *C. ovalis* demonstrated a remarkable ability to prevent hemolysis, approaching the efficacy of the standard. A study represented that hexadecanoic acid, as evidenced by its stable binding within the active site of phospholipase A2 (PLA2), demonstrates a potential anti-inflammatory effect by impeding substrate entry and hindering catalytic residues. Understanding its interaction with PLA2 may contribute to the development of drugs targeting chronic inflammatory conditions such as rheumatoid arthritis and asthma (Aparna et al., 2012). So, our finding suggests that the presence of high amount of hexadecanoic acid is the reason for the extract potential membrane-stabilizing effect.

Using the tail immersion test, we assessed the central analgesic ability of the crude extract of *C. ovalis*. This procedure results in supra-spinal pain that is centrally mediated. While it is generally established that acetic acid somehow triggers the release of endogenous pain mediators, activating the pain-sensing neurons (Arslan and Bektas, 2010), in this study, the peripheral analgesic activity of the crude methanolic extract from the leaves of *C. ovalis* was determined by inducing writhing on mice with acetic acid.

This study unveiled a statistically significant and noteworthy central analgesic effect from the crude methanol leaf extract of *C. ovalis*, which was both dose- and time-dependent. Interestingly, the moderate dose (400 mg/kg) exhibited the most potent peripheral analgesic activity, with statistically significant results, followed by the highest dose (600 mg/kg). This finding could serve as an initial avenue for further investigation aimed at uncovering more precise insights into the plant’s pain-relieving properties.

To better understand the biological roles of substances from nature and to estimate ligand–receptor interactions, molecular docking studies were widely used. It offers further details on potential methods of action and binding style in the binding sites of certain proteins (Alam et al., 2020b).

As a matter of fact, a molecular docking investigation was carried out to clarify and validate the experimental findings from biological research. This technique confirms the experimental results. In order to find the central and peripheral analgesic properties, the identified compounds docked against Mu (PDB ID: 5C1M) and COX-2 (PDB ID: 1CX2) receptors, respectively, while TNF-α (PDB ID: 2AZ5) and TPA (PDB ID: 1A5H) receptors were selected for screening the anti-inflammatory and thrombolytic activities of identified compounds from the crude methanolic extract of leaves of *C. ovalis.*

The strongest affinity toward the Mu receptor was observed by C30 or trispiro[4.2.4.2.4.2.]heneicosane (−8.8 kcal/mol), followed by C34 or dihydroartemisinin, 10-O-(t-butyloxy)- (−8.5 kcal/mol). Meanwhile, distearin (C27), dibenzo[b,f][1,4]diazocine, 5,6,11,12-tetrahydro-5-methyl- (C7), and 4aH-cycloprop[e]azulen-4a-ol, decahydro-1,1,4,7-tetramethyl-, [1aR-(1a.alpha.,4.beta.,4a.beta.,7. alpha.,7a.beta.,7b.alpha.)]- (C39) suppressed the activity of standard (−7.9 kcal/mol) against the COX-2 receptor with binding affinities of −9.3, −9, and 8.4 kcal/mol, respectively. Subsequently, trispiro[4.2.4.2.4.2.]heneicosane (C30) and dibenzo[b,f][1,4]diazocine, 5,6,11,12-tetrahydro-5-methyl (C7) illustrated remarkable binding affinities toward TNF-α and TPA (Table 4).

The fact that C7 and C36 have higher binding affinities than projections for each of these four receptors suggests that they could be future therapeutic options for these various disorders. However, C27 exhibited strong affinity against COX-2, TNF-α, and TPA receptors, while C30 and C34 manifested lower binding affinity against Mu, TNF- α, and TPA receptors. In addition, C39 was found to have strong binding activities against Mu, COX-2, and TNF- α receptors.

Tables 5–7 show the various ADME/T parameters of the most prominent compounds found through the *in silico* study. According to Lipinski, a substance will be orally accessible if it meets the following requirements: molecular weight <500 amu, hydrogen bond donor sites <5, hydrogen bond acceptor sites <10, and lipophilicity value LogP ≤5 (Zhang and Wilkinson, 2007). The above data demonstrated that C6 and C36 did not violate any of Lipinski’s five principles, but C3, C7, C28, C29, and C30 were determined to have violated one of Lipinski’s laws. Additionally, C9, C15, C21, C22, C34, and C39 each had two violations. Finally, C26 and C27 demonstrated three violations of Lipinski’s law. The results indicate that the compounds adhere to Lipinski’s criteria, which means they are safe for oral use and may function as promising medication candidates.

**TABLE 5:** Absorption and distribution profile of the selected compounds which showed the best interaction against these receptors.

Properties	Absorption							Distribution			
Model name (unit)	Water solubility (log mol/L)	Caco2 permeability (log Papp in 10–6 cm/s)	Intestinal absorption (human) (% absorbed)	Skin permeability (log Kp)	P-glycoprotein substrate	P-glycoprotein I inhibitor	P-glycoprotein II inhibitor	VDss (human) (log L/kg)	Fraction unbound (human) (fu)	BBB permeability	CNS permeability
C3	−4.36	1.513	97.394	−2.654	No	Yes	No	−0.762	0.048	−0.106	−2.05
C6	−2.522	1.547	95.713	−1.236	No	No	No	0.325	0.362	0.409	−1.677
C7	−2.331	1.506	74.539	−2.061	No	No	No	0.49	0.19	0.169	−1.336
C9	−3.876	1.668	92.254	−2.364	No	No	No	0.545	0.042	0.47	−0.858
C15	−6.455	1.644	93.941	−2.255	No	No	No	0.311	0.07	0.725	−1.672
C21	−6.849	1.643	94.049	−2.434	No	No	No	0.333	0.031	0.744	−1.621
C22	−8.558	1.37	89.328	−2.793	No	No	Yes	0.579	0	1.033	−1.144
C26	−4.141	1.625	94.593	−1.662	No	No	No	0.183	0.336	0.606	−2.201
C27	−3.907	−0.108	84.821	−2.735	Yes	No	Yes	−0.956	0.171	−1.044	−3.112
C28	−3.195	1.482	93.208	−3.125	Yes	No	No	0.563	0.552	0.585	−3.228
C29	−2.009	1.39	89.429	−2.735	No	No	No	0.012	0.807	0.276	−3.186
C30	−7.358	1.441	93.087	−2.546	No	No	Yes	0.73	0	0.797	−1.366
C34	−3.651	1.271	83.834	−2.735	Yes	No	No	−1.374	0.209	0.672	−1.239
C36	−2.8	0.108	66.393	−3.045	No	No	No	−0.294	0.67	−0.819	−3.198
C39	−3.98	1.496	93.979	−2.247	No	No	No	0.571	0.326	0.617	−2.536

**TABLE 6:** Metabolism and excretion profile of the selected compounds which showed the best interaction against these receptors.

Properties	Metabolism							Excretion	
Model name (unit)	CYP2D6 substrate	CYP3A4 substrate	CYP1A2 inhibitor	CYP2C19 inhibitor	CYP2C9 inhibitor	CYP2D6 inhibitor	CYP3A4 inhibitor	Total clearance (log mL/min/kg)	Renal OCT2 substrate
C3	No	Yes	No	No	No	No	No	0.409	No
C6	No	No	No	No	No	No	No	0.254	No
C7	No	Yes	Yes	No	No	No	No	0.498	Yes
C9	No	Yes	Yes	No	No	No	No	0.781	No
C15	No	No	Yes	No	No	No	No	1.563	No
C21	No	Yes	Yes	No	No	No	No	1.594	No
C22	No	Yes	Yes	No	No	No	No	2.033	No
C26	No	No	No	Yes	No	No	No	1.645	No
C27	No	Yes	No	No	No	No	No	2.239	No
C28	No	No	No	No	No	No	No	0.419	No
C29	No	No	No	No	No	No	No	−9.401	No
C30	No	Yes	Yes	No	No	No	No	0.841	No
C34	No	No	Yes	Yes	No	No	No	0.518	No
C36	No	No	No	No	No	No	No	0.32	No
C39	No	No	No	Yes	Yes	No	No	0.856	No

**TABLE 7:** Toxicology and drug-likeliness profile of the selected compounds which showed the best interaction against these receptors.

Properties	Toxicity										Drug-likeness	
Model name (unit)	AMES toxicity	Max. tolerated dose (human) (log mg/kg/day)	hERG I inhibitor	hERG II inhibitor	Oral rat acute toxicity (LD50) (mol/kg)	Oral rat chronic toxicity (LOAEL) (log mg/kg_bw/day)	Hepatotoxicity	Skin sensitization	*Tetrahymena pyriformis* toxicity (log ug/L)	Minnow toxicity (log mM)	Lipinski’s rule of five	Bioavailability score (%)
C3	No	−0.62	No	No	2.531	0.87	No	No	0.534	−0.262	No; 1 violation: MW > 350	0.55
C6	No	0.921	No	No	1.841	2.168	No	No	−0.022	1.31	Yes	0.55
C7	Yes	0.096	No	No	1.702	0.769	No	No	1.678	1.101	No; 1 violation: MW < 250	0.55
C9	No	0.409	No	No	2.346	1.736	No	Yes	1.667	−0.108	No; 2 violations: MW < 250, XLOGP3>3.5	0.55
C15	No	0.432	No	No	1.643	2.76	No	Yes	2.302	−1.221	No; 2 violations: rotors>7, XLOGP3>3.5	0.55
C21	No	0.386	No	No	1.633	2.833	No	Yes	2.172	−1.536	No; 2 violations: rotors>7, XLOGP3>3.5	0.55
C22	No	−0.06	No	Yes	1.611	1.146	No	Yes	0.748	−2.613	No; 2 violations: rotors>7, XLOGP3>3.5	0.55
C26	No	0.43	No	No	1.693	2.392	No	Yes	1.629	0.192	No; 3 violations: MW < 250, rotors>7, XLOGP3>3.5	0.55
C27	No	0.142	No	No	3.105	0.496	No	No	0.285	−6.303	No; 3 violations: MW > 350, rotors>7, XLOGP3>3.5	0.17
C28	No	0.184	No	No	2.027	0.754	No	Yes	0.286	1.5	No; 1 violation: XLOGP3>3.5	0.55
C29	Yes	0.319	No	No	2.482	5.273	No	No	0.285	6.004	No; 1 violation: MW < 250	0.56
C30	No	−0.25	No	Yes	1.631	1.562	No	No	1.021	−1.112	No; 1 violation: XLOGP3>3.5	0.55
C34	Yes	1.375	No	No	2.482	0.188	No	No	0.285	4.787	No; 2 violations: MW > 350, XLOGP3>3.5	0.55
C36	No	1.065	No	No	2.389	1.784	Yes	No	0.252	2.593	Yes	0.55
C39	No	0.215	No	No	1.698	1.185	No	Yes	1.118	1.105	No; 2 violations: MW < 250, XLOGP3>3.5	0.55

However, all of the substances in Table 7 with the exception of C27, which had a bioavailability value of 0.17%, showed a bioavailability score of 0.55%. Consequently, pkCSM is a unique method for predicting pharmacokinetic and toxicological effects that use graph-based signatures to reflect the chemistry and topology of small molecules (Pires et al., 2015). None of these compounds except C34 was found to be hepatotoxic. However, none of these compounds inhibits hERG I, and except for C22 and C30, none of them inhibits hERG II, indicating these compounds may not have cardiotoxicity (Muster et al., 2008). In addition, all of these compounds displayed negative values for water solubility (log mol/L), indicating their lipophilic nature, which facilitates efficient absorption (Table 5).

All these assumptions from the ADME/T study influenced the idea of treating these compounds, particularly which exhibited prominent binding affinities toward the abovementioned receptors, for their drug-like candidacy and further investigation.

In this study, the presence of the aforementioned substances, which showed potential binding affinities against those four receptors, supported the outcomes of biological activities such as thrombolytic and membrane-stabilizing as well as peripheral and central analgesic effects of the *C. ovalis* leaf extract. In addition, this docking suggested that those compounds may become potential drug candidates for these four disease states. However, in order to identify and develop the pharmaceutically active constituent from the extracts of *C. ovalis*, extensive investigations are needed to investigate the real mechanism of action and drug safety concerns.

## 5 Conclusion

According to the findings of this study, *C. ovalis* leaves can be a notable source of analgesic and membrane-stabilizing potentials as well as a viable candidate for thrombolytic potential. In addition, several bioactive potential constituents showed favorable binding affinity to particular proteins in molecular docking analysis, and the ADME/T investigation demonstrated their pharmacokinetics and drug-like characteristics. As a result, the computational work has confirmed the biological activity testing data and offered encouraging insight for evaluating *C. ovalis* as a noteworthy therapeutic candidate. Further investigation needs to be carried out to isolate these compounds and establish their mechanism of action against these specific receptors in a broad setup.

## Data Availability

The raw data supporting the conclusion of this article will be made available by the authors, without undue reservation.
